# Predicting antibiotic consumption in internal medicine departments: a derivation and validation study of patient-related factors

**DOI:** 10.1017/ash.2026.10803

**Published:** 2026-09-14

**Authors:** Zvi Shimoni, Talya Finn, Paul Froom

**Affiliations:** 1 The Adelson School of Medicine, Ariel University, Ariel, Israel; 2 Sanz Medical Center, Laniado Hospital, Netanya, Israel; 3 Department of Infectious Disease, Sanz Medical Center, Laniado Hospital, Netanya, Israel; 4 Clinical Utility Department, Sanz Medical Center, Laniado Hospital, Netanya, Israel; 5 Gray Faculty of Medical and Health Sciences, University of Tel Aviv, Tel Aviv, Israel

## Abstract

**Background:**

Identifying non-prescriber-related factors related to antibiotic consumption is a critical first step to increasing confidence in surveillance results and targeting audit and feedback interventions.

**Methods:**

This study analyzed data from 11,402 patients hospitalized across 3 internal medicine departments between January 2020 and November 2021. The outcome variable was the proportion of patients treated and the daily days of therapy (DOT) per 100 hospitalization days (HD). Independent variables included demographic data, admission laboratory results, nursing assessments (dementia, bedridden status), a chief complaint of fever, bacteriuria, and a discharge diagnosis of infection. A multivariate logistic regression model was developed using 2020 data and validated with 2021 data to determine factors associated with antibiotic consumption.

**Results:**

The cohort had a mean age of 73 ± 18 years, and 43.0% of patients received antibiotics, resulting in a consumption rate of 51.8 DOT per 100 HD. Significant predictors included a discharge diagnosis of infection, chief complaint of fever, elevated white blood cell count and neutrophil proportion, bacteriuria, and short or prolonged hospital length of stay. The model in 2020 was confirmed in 2021 and achieved a C-statistic of 0.907 (0.902–0.912).

**Conclusions:**

Adjusting for non-modifiable patient factors establishes a more accurate baseline for surveillance, increases the reliability of clinical performance audits, and suggests targets for intervention. Although the model demonstrated high predictive power, these results require validation in other clinical settings.

## Introduction

Antibiotic resistance is a growing global health concern with significant implications for patient outcomes and healthcare costs. Understanding the predictors of antibiotic use in internal medicine departments is crucial for developing effective stewardship programs to optimize prescribing practices. Although several strategies may be employed to achieve prudent antibiotic use in hospitals, an initial step should be identifying non-prescriber-related factors; this can increase confidence in surveillance results and help target specific areas for intervention.^1^ Audit and feedback, defined as a summary of clinical performance over a specified period, is a recommended intervention^2^; thus, knowing the contributors to antibiotic use is essential for this purpose. However, few studies have examined factors associated with hospital-based antibiotic use, particularly regarding internal medicine patients presenting to the emergency department with acute symptoms.^1,3–5^

In this study, we selected patients hospitalized in 3 internal medicine departments to determine factors associated with actual antibiotic consumption. We also investigated the potential effects of reducing use in patients without a primary diagnosis of infection, those with short hospital stays (as a proxy for divertible hospitalizations,^6^ and cases of prolonged antibiotic use (>7 d) where the days of antibiotic therapy might have been shortened.

## Methods

All patients hospitalized in 1 of 3 internal medicine departments between January 1, 2020, and November 1, 2021, were selected, with data obtained from an electronic database. Patients with Coronavirus disease were segregated and not hospitalized in those departments. Antibiotics administered were recorded by nursing staff. The metrics used were the proportion of patients treated and the total number of daily drugs given per 100 hospitalization days (DOT per 100 HD). If a patient received 2 drugs on the same day, the count for that day was recorded as 2. Antibiotics designated for limited use included aminoglycosides, carbapenems, colistin, and piperacillin/tazobactam.

Independent variables included age, gender, previous hospitalization within the past 28 days, admission nursing assessments (dementia, bedridden status), chief complaint of fever, a positive urine culture, admission with a permanent catheter, admission laboratory test results, and a discharge diagnosis of infection (yes/no). A diagnosis of bacterial infection on discharge included either the ICD code or a written explanation of urinary tract infection, bacterial respiratory tract infection, pneumonia, skin infection, abscess, acute bronchitis, bacteremia/sepsis, cholecystitis, bronchiectasis, endocarditis, otitis media, osteomyelitis, meningitis, neutropenic fever, or pseudomembranous colitis. Laboratory markers at admission included total white blood cell (WBC) counts (≤12, 12.1–14.9, ≥15 × 10^9^ cells/L), proportion of neutrophils (<70%, 70%–89%, and 90% or more), serum albumin (<3, 3–3.49, ≥3.5 g/dL), hemoglobin (<10, 10–11.9, ≥12 g/dL), platelet counts (<100, 100–149, ≥150 × 10^9^ cells/L), and serum creatinine (<1, 1–1.9, ≥2 mg/dL). There were only a few missing data points for the laboratory tests that were assumed to be in the normal range. The categories were established before the analysis, and binned similarly to that reported previously for predicting in-hospital mortality.^7^

### Statistical analysis

We calculated the DOT per 100 HD and the proportion of various antibiotics administered (Table 1). We determined the proportion of patients treated over 7 days, those treated with antibiotics, and discharged within 2 days, and those without a diagnosis of infection on discharge. Also, we determined the proportion of patients treated with antibiotics that are limited (see above). Univariate and multivariate logistic regression analysis determined the odds ratios and 95% confidence intervals (OR, 95% CI) of the independent variables’ association with the outcome variables. Multivariate analysis included all variables that were retained if they significantly added to the model. The others were entered one at a time and retained if significant. We used all variables in a model intended for retrospective risk adjustment. Then we included only variables available on admission to predict antibiotic use at admission. The development model was performed for the year 2020 and then validated using data from 2021. The independent variables included were those that added significantly to the model during both years. The Hosmer–Lemeshow (H–L) was calculated, and deciles of risk were presented. The C-statistic was calculated for the entire study period.

**Table 1.** Drugs dispensed in patients hospitalized Table 1 long description.

Drug	N	All patients N=11,402 (%)	Treated with antibiotics N=4,903 (%)
Other	415	3.6	8.5
Metronidazole	1,036	9.1	21.1
Carbapenem	355	3.1	7.2
Quinolone	256	2.2	5.2
Aminoglycosides	229	2.0	4.7
Penicillin	157	1.4	3.2
Beta lactams	206	1.8	4.2
Macrolide	413	3.6	8.4
Cephalosporins – other	1,280	11.2	26.1
Ceftriaxone	2,840	24.9	57.8
Chloramphenicol	826	7.2	16.8
Clindamycin	540	4.7	11.0
Colistin	9	0.08	0.1
Vancomycin	120	1.1	2.4
Piperacillin/tazobactam	470	4.1	9.6
Tetracycline	629	5.5	11.5
Reserve antibiotics	1,015	8.9	20.7

Ethical committee approval was not required because the study was conducted as part of the quality control program without patient identifiers.

## Results

The study included 11,402 internal medicine patients with a mean age of 73 ± 18 years. Females comprised 50.9% of the cohort, and the mean hospital stay was 4.6 ± 5.9 days. A total of 4,903 patients (43.0%) received antibiotics, resulting in 27,001 DOT and a consumption rate of 51.8 DOT per 100 HD. Cephalosporins were the most common antibiotics administered, led by ceftriaxone (Table 1), which was given to over 50% of treated patients. Limited antibiotics were dispensed to 20.7% of treated patients and vancomycin to 2.4%. There were 7.1% (815/11402) of the cohort who received antibiotics and were hospitalized for ≤48 hours, accounting for 4.7% of the DOT (n = 1,270). There were 196 patients (1.7% of the patients) with over 7 days of antibiotic therapy who had 3,661 DOT (13.6%), and stopping treatment after 7 days would have reduced total antibiotic consumption by 917 DOT (3.4%). There were 1,686 patients treated with antibiotics without a primary diagnosis of infection who had a total of 4,238 DOT (15.7%). Thus, potentially up to 23.8% of the DOT could be prevented if no patients were treated over 7 days of antibiotics, not treated if they were able to be discharged within 2 days, and did not receive antibiotics if they didn’t have a primary diagnosis of infection. Almost all antibiotic treatments were initiated upon admission, with only 0.4% of the patients (n = 40) starting antibiotics after the third day of hospitalization.

### Associations with antibiotic therapy

All the independent variables were associated with antibiotic therapy (Tables 2 and 3). Variables that significantly added to the model included a chief complaint of fever, short or prolonged hospital stay, elevated white counts and proportion of neutrophils, thrombocytopenia, and bacteriuria. Department C had a lower odd of antibiotic therapy compared to the other 2 departments.

**Table 2.** Univariate analysis variables associated with antibiotic therapy Table 2 long description.

Variable		N	N (%)	OR	95% CI
Age years	< 60	2,371	848 (35.8)	1.32	1.26–1.38
	60–79	4,081	1,647 (40.4)		
	≥80	4,950	2,408 (48.6)		
Female	Y	5,801	2,442 (42.1)	0.93	0.86–1.0
	N	5,601	2,461 (43.9)		
Previous hospitalization	Y	1,373	626 (45.6)	1.13	1.01–1.26
	N	10,029	4,277 (42.6)		
Fever	Y	1,324	1,120 (84.6)	9.14	7.83–10.66
	N	10,078	3,783 (37.7)		
Bedridden	Y	2,942	1,805 (61.4)	2.75	2.52–3.00
	N	8,460	3,098 (36.6)		
Dementia	Y	1,853	1,153 (62.2)	2.55	2.30–2.82
	N	9,549	3,750 (39.3)		
Permanent urinary	Y	616	448 (72.7)	3.79	3.16–4.54
Catheter	N	10,786	4,455 (41.3)		
Hospital days	>7	1,563	1,159 (74.2)	4.67	4.14–5.27
	3–7	5,150	2,929 (56.9)		
	≤2	4,689	815 (17.4)	0.14	0.12–0.15
Infectious disease	N	7,721	1,639 (21.2)	29.05	25.87–32.61
	Y	3,681	3,264 (88.7)		
Department	A	3,521	1,554 (44.1)	1.07*	0.99–1.16
	B	4,116	1,793 (43.6)	1.10**	1.00–1.20
	C	3,765	1,556 (41.3)	0.90	0.83–0.98

Note. CI, confidence interval; OR, odds ratio.

**Table 3.** Univariate analysis laboratory tests associated with antibiotic therapy Table 3 long description.

Variable		N	N (%)	Odds ratio	95% CI
Albumin (g/dL)	≥ 3.5	9,151	3,465 (37.9)	2.04	1.90–2.18
	3–3.3	1,471	926 (63.0)		
	<3.0	780	512 (65.5)		
Creatinine (mg/dL)	< 1.0	5,012	1,969 (39.3)	1.21	1.14–1.27
	1.0–1.9	4,969	2,266 (45.6)		
	≥2.0	1,421	668 (47.0)		
Glucose (mg/dL)	< 150	7,730	3,191 (41.3)	1.16	1.10–1.22
	150–199	1,994	901 (45.2)		
	≥200	1,678	811 (48.3)		
WBC (10^9^/L)	< 12	8,179	2,774 (33.9)	2.39	2.26–2.53
	12–14.9	1,477	835 (56.5)		
	≥ 15	1,746	1,294 (74.1)		
Neutrophils (%)	< 70	4,216	1,114 (24.1)	2.29	2.19–2.39
	70–79	3,298	1,322 (40.1)		
	80–89	2,959	1,828 (61.8)		
	≥90	926	739 (79.5)		
Hemoglobin (g/dL)	≥ 12	6,497	2,558 (39.4)	1.24	1.18–1.30
	10–11.9	3,244	1,545 (47.6)		
	<10	1,661	800 (48.2)		
Platelets (× 10^9^/L)	≥ 150	10,150	4,212 (41.7)	1.42	1.30–1.55
	100–149	957	497 (51.9)		
	<100	340	194 (57.1)		
Positive urine culture	Y	10,223	3,939 (38.5)	7.15	6.14–8.34
	N	1,179	964 (81.8)		

Note. CI, confidence interval; WBC, white blood cell.

Models predicting associations with antibiotic use, use of antibiotics that are limited, and antibiotic use in those hospitalized for up to 48 hours, treatment >7 days, and in those without a final diagnosis of an infection.

The model in 2020 predicting the association with antibiotic use was confirmed in 2021 (Table 4). The H–L statistic was significant (*P* < .001) showing that the model systematically over-or underestimated risk at certain probability levels. Nevertheless, the model resulted in a graded increase in risk and might be usable clinically even though the risk did not increase linearly (Table 4). The combined model was excellent, with a C-statistic of 0.907 (0.902–0.912). Department C did not add significantly to the model (1.00, 0.91–1.11).

**Table 4.** Factors associated with antibiotic treatment Table 4 long description.

Predictors	2020	2021	Both years
Infections	16.94 (14.21–20.19)	18.60 (15.27–22.66)	17.48 (15.34–19.92)
Fever^*^	2.17 (2.05–3.58)	2.47 (1.83–3.34)	2.61 (2.13–3.20)
LOS^*^ <3 d	0.21 (0.18–0.25)	0.19 (0.16–0.22	0.20 (0.18–0.23)
LOS^*^ >7 d	1.39 (1.13–1.71)	2.20 (1.70–2.84)	1.67 (1.42–1.96)
WBC^*^	1.48 (1.33–1.65)	1.70 (1.50–1.91)	1.58 (1.45–1.71)
Neutrophils^*^	1.39 (1.28–1.51)	1.54 (1.40–1.70)	1.45 (1.37–1.55)
Platelet^*^	1.44 (1.22–1.70)	1.29 (1.08–1.54)	1.37 (1.21–1.55)
Creatinine^*^	0.91 (0.82–1.01)	0.86 (0.77–0.97)	0.89 (0.82–0.96)
Bacteriuria	2.15 (1.62–2.84)	2.15 (1.58–2.91)	2.14 (1.75–2.63)
Deciles of risk			
1–2	51/1,149 (4.4%)	39/1,081 (3.4%)	97/2,274 (4.3%))
3–4	171/1,318 (13.0%)	137/1,112 (12.3%)	300/2,396 (12.5%)
4–6	392/1,205 (32.5%)	281/971 (28.9%)	682/2,184 (31.2%)
7–8	904/1,219 (74.2%)	773/1,068 (73.4%)	1762/2,264 (77.8%)
9–10	1143/1,221 (93.6%)	1012/1,058 (95.7%)	2158/2,285 (94.4%)
H–L statistic^*^	*P* < 0.001	*P* = 0.0468	*P* < 0.001

Note. LOS, length of stay; H–L, Hosmer–Lemeshow statistic; WBC, white blood cell.

* Chief complaint – fever. See Tables 2 and 3 for categories.

The model considering only admission variables was also good with a C-statistic of 0.838 (Table 5). In this model, the variables included those associated with patient frailty (being bedfast, demented, with lower hemoglobin and albumin admission values). Patients in Department C had a lower odd of antibiotic treatment in this model, 0.88 (0.80–0.97).

**Table 5.** Admission variables associated with antibiotic therapy Table 5 long description.

Predictors	2020–2021
Hospitalized in last 4 wk	0.82 (0.71–0.95)
Admission infectious diagnosis	
Urinary tract	10.42 (8.15–13.32)
Respiratory tract	9.57 (7.60–12.06)
Skin/soft issue	13.65 (10.59–17.58)
Other	3.63 (2.23–5.90)
Bedfast	1.60 (1.42–1.80)
Dementia	1.52 (1.32–1.74)
Fever^*^	5.15 (4.33–6.13)
WBC^*^	1.64 (1.53–1.75)
Neutrophils^*^	1.66 (1.57–1.75)
Platelet^*^	1.49 (1.34–1.65)
Hemoglobin	1.07 (1.00–1.14)
Albumin^*^	1.37 (1.25–1.50)
Department C	0.88 (0.80–0.97)
Deciles of risk	
1–2	230/2,272 (10.1%)
3–4	436/2,290 (19.0%)
5–6	778/2,279 (34.1%)
7–8	1,440/2,298 (62.7%)
9–10	2,019/2,263 (89.2%)
Hosmer–Lemeshow statistic	<0.001
C-statistic	0.838 (0.831–0.845)

Note. WBC, white blood cell.

* Fever is the chief complaint, see Tables 2 and 3 for categories.

Factors that predicted antibiotic therapy during a hospitalization of 48 hours or less included being febrile, being of a younger age, not being bedfast, without a permanent urinary catheter, higher albumin concentrations, and lower glucose and creatinine admission blood tests after adjustment for an admission diagnosis of infection. The model was poor but demonstrated a graded non-linear association of risk (Table 6). None of the departments added significantly to the model.

**Table 6.** Factors associated with antibiotic treatment and discharge within 48 hours Table 6 long description.

Predictors	Odds ratio	95% CI
Age group	0.67	0.61–0.75
Bedfast	0.67	0.54–0.83
Urinary catheter	0.65	0.43–0.98
Fever	1.30	1.06–1.60
Admission infectious diagnosis		
Urinary tract	2.42	1.88–3.10
Respiratory tract	1.97	1.53–2.53
Skin/soft tissue	2.71	2.11–3.50
Other	2.73	1.56–4.79
WBC	1.25	1.13–1.38
Neutrophil %	1.18	1.08–1.28
Glucose	0.82	0.74–0.92
Creatinine	0.83	0.73–0.93
Albumin	0.84	0.71–0.98
Deciles of risk		
1–2	73/2,273 (3.2%)	
3–4	74/2,287 (3.2%)	
5–6	110/2,281 (4.8%)	
7–8	175/2,281 (7.7%)	
9–10	383/2,280 (16.8%)	
Hosmer–Lemeshow statistic	<0.001	
C-statistic	0.698 0.689–7.06	

Note. CI, confidence interval; WBC, white blood cell.

The model (not shown) for the associations with antibiotic therapy in those without a diagnosis of infection was poor, with a C-statistic of 0.609 (0.600–0.618) and included associations with patient fragility (low albumin, bedfast, low hemoglobin, and low platelet counts) as well as an increased WBC and neutrophil proportions. Increased odds were found for department A and lower odds in department B after all significant variables were included, 1.15 (1.13–1.41) and 0.84 (0.76–0.94), respectively.

Of those receiving antibiotics, Departments A, B, and C extended the treatment over 7 days in 3.9% (61/1554), 3.0% (54/1793), and 5.2% (81/1556), respectively. (*P* = .0053). Unadjusted odds ratio for Department C was 1.77 (1.25–2.51), and after adjustment for increased age, being bedfast, a low serum albumin, a high proportion of neutrophils, and not having a urinary tract infection, Department C still significantly added to the model with an OR of 1.54 (1.15–2.07). The H–L statistic was not significant, and the model was poor (C-statistic = 0.693 (0.679–0.705).

There were 1,015 (8.9%) of the patients who received limited antibiotics. The model for antibiotics with limited indications was different from the model for any treatment with antibiotics (Table 7). Dementia, permanent catheter, and previous hospitalization added to the model, whereas creatinine, white blood counts, neutrophils, and platelets did not add to the model. The model was fair with a C-statistic of 0.759 (0.751–0.767). Department B administered more antibiotics with limited use compared to the other Departments.

**Table 7.** Admission diagnosis factors associated with treatment of antibiotics that are limited Table 7 long description.

Predictors	Odds ratio	95% CI
Female	0.82	0.71–0.94
Previous hospitalization^*^	1.22	1.00–1.47
Permanent catheter	2.21	1.77–2.75
Bedfast	1.48	1.27–1.73
Fever	2.21	1.87–2.62
UTI admission diagnosis	5.14	4.27–6.18
WBC	1.14	1.04–1.25
Neutrophils proportion	1.16	1.08–1.26
Hemoglobin	1.40	1.27–1.53
Platelet counts	1.37	1.20–1.57
Department B	1.41	1.23–1.63
Deciles of risk		
1–2	54/2152 (2.5%)	
3–4	88/2416 (3.6%)	
5–6	116/2266 (5.1%)	
7–8	199/2289 (8.7%)	
9–10	558/2279 (24.5%)	
Hosmer–Lemeshow statistic	0.043	
C-statistic	0.759 0.751–0.767	

Note. CI, confidence interval; UTI, urinary tract infection; WBC, white blood cell.

* Previous hospitalization in the last 28 days.

## Discussion

The primary finding of this study is that antibiotic use is strongly associated with a discharge diagnosis of infection, factors associated with infection (chief complaint of fever, elevated WBC counts, higher proportion of neutrophils, bacteriuria), and duration of hospitalization. The model was excellent, with a C-statistic of 0.907, and the model, although not linear, had the lowest and highest 2 deciles of risk of 4.3% and 94.4%, respectively. This model might be adopted to compare antibiotic use in various settings.

However, several predictors in this model such as the discharge diagnosis of infection at discharge and length of stay categories might be influenced by coding and downstream clinical decisions, so we included models that considered only variables available on admission. The model predicting antibiotic use was good, and there was also a graded risk, with the lowest and highest 2 deciles of risk of 10.1% and 89.2%, respectively. The model identified 1 of the 3 internal medicine departments with a lower odd of using antibiotics.

There are limited attempts to predict antibiotic use in the hospital, but the results of studies with differing methodology and inclusion criteria are mostly consistent with our results. A study in Norwegian hospitals found that the hospital’s antibiotic use was associated with short and long hospital stays, ICD-10 infectious codes, and more nurse availability.^1^ A study across 576 US hospitals found that 65% of patients received antibiotics, which was associated with geographic location, a bacterial infection diagnosis, and younger age.^8^ A study in Israel in 2003–2004 of 26 internal medicine departments reported that the only variables significantly contributing to predicting the defined daily doses (DDD) per 100 bed-days were hospital affiliation, which explained 43% of the variance, and the rate of 1-day hospitalizations, which explained only 7.4% of the variance.^9^ Variables not contributing to the model included age, gender, diagnosis of infectious diseases, neutropenia, respiratory support, and the Charlson score. We found that those hospitalized for up to 48 hours had a lower prevalence of antibiotic therapy, consistent with an increased wait-and-see approach in patients who might not have required hospitalization. Poran et al found that antibiotic use after 48 hours from admission, prescribed in 8.1% of patients, was associated with male gender, poor functional capacity, prophylactic antibiotic use, transplantation, urinary catheterization, the use of a central venous catheter, poor mental status, and low albumin. The model was only fair with a C-statistic of 0.72.^3^ Only 0.4% of patients in this study had antibiotic treatment starting more than 48 hours after admission, and although male gender, dementia, low albumin, and permanent catheter were associated with antibiotic therapy, they did not add to the multivariate analysis. Beck et al studied patients from mostly hematological, oncological, and cardiothoracic surgery departments selected with planned or high likelihood of receiving systemic antibiotic treatment for ≥5 days within the first 10 days of their stay; 52.8% received antibiotics, and they found that key predictors included hematologic malignancies, immunosuppressive medication, and past hospitalizations with a C-statistic of 0.74.^4^ We also found that previous hospitalization in the 28 days predicted the use of antibiotics that are limited but had a lower odds ratio for the association with any antibiotic therapy. In a systematic review, Lim et al found that patients seen in the emergency department with normal C-reactive protein levels and positive influenza tests were less likely to receive antibiotic treatment.^5^ Nonclinical factors associated with antibiotic use were longer waiting time and perceived patient desire for antibiotics. Patients attended by internal medicine physicians co-managed by house staff or who visited an Emergency Department that provided education to healthcare providers on antibiotic use were less likely to receive antibiotics. We did not study those variables. Clearly, studies of internal medicine patients in other settings are needed to confirm our findings.

A secondary finding of our study was that nearly a quarter of the total antibiotic days were in patients with short hospital stays, antibiotic treatment over 7 days, and in those without a primary diagnosis of infection. Selecting those outcomes might aid in comparing internal medicine departments and focus audits and feedback interventions. The models predicting those outcomes were not as strong as for associations with antibiotic use, but nevertheless identified differences between the Departments that might aid in focusing attempts to change antibiotic prescribing behavior.

Short hospital stays of under 48 or 72 hours may suggest that a patient could have been cared for in an alternative setting, such as a high-acuity clinic.^6,10,11^ Also delayed antibiotic prescription, using a wait-and-see approach with re-evaluation of the patient, has been reported as a useful tactic to help reduce antibiotic use, particularly for respiratory tract infections.^12^ A before-and-after trial compared universal early antibiotic therapy (aggressive approach) to a second period when only hypotensive patients received immediate antibiotic therapy.^13^ After the change in policy, patients underwent therapy only after microbiological confirmation of infection and had a higher rate of initial appropriate therapy, shorter duration of therapy, and lower mortality. Further studies are warranted to determine when postponing treatment is safe and can reduce hospital antibiotic use. Chart reviews are required to determine if in-hospital systemic antibiotic therapy might be reduced in this group of patients.

Of the total drug days, 13.6% were in patients with over 7 days of antibiotic therapy, and 3.4% of the drug days occurred after day 7 that might have been excessive. The proportion of inappropriate antibiotic therapy days is unclear, but for most infections, treatment should be limited to 5 days or less. For example, the American College of Physicians recommends that treatment for chronic obstructive lung disease exacerbations and community-acquired pneumonia be limited to 5 days.^14^ In US hospitals, unsupported treatment was found for community-acquired pneumonia in 79.5%, most commonly for excess duration.^15^ Chart review is required to determine the proportion of days that were not indicated in that group and those receiving antibiotics without a primary diagnosis of a bacterial infection.

This study has several strengths. We selected a homogeneous cohort of internal medicine patients, excluding elective admissions and hospitalization in intensive care, cardiology, and surgical departments, increasing internal validity. Antibiotic use was based on consumption rather than prescription. The exploratory model was confirmed during the subsequent year, and the final model was excellent.

There are also limitations. The study was done in a single hospital, and extrapolation to other settings should be done with caution. Even for internal medicine departments, studies are needed to confirm our results because it is unclear if variable usage rates will affect the observed associations. However, we found the rate of total drug days of treatment to be 51.8 DOT per 100 HD, consistent with the findings in 5 internal medicine departments in Canada with usage rates that ranged from 45.4 to 58.1 DDD per 100 HD.^16^ Also, a systematic review of antibiotic consumption in acute care hospital medical departments reported use of 59.3 per 100 hospital days in studies where collection methods were clearly reported.^17^ Still in that review, methods of data collection were significantly associated with consumption rates. The factors included data sources (dispensing vs purchase vs usage data), the counting of admission and discharge days, and inclusion of low-consumption departments. They concluded that major heterogeneity precluded defining acceptable antibiotic consumption ranges in acute care hospitals. There is also significant international variation in the consumption of antibiotics with limited indications that may be an important consideration when extrapolating our results to other settings. For example, in our hospital, only 4.1% of patients were treated with piperacillin/tazobactam, and 1.1% with anti-MRSA(methacillin-resistant Staphylococcus aureus) treatment, whereas in US hospitals, it was 14.5% and 23.9%, respectively.^8^ Second, we did not do chart reviews, so we cannot determine acceptable antibiotic consumption ranges in internal medicine departments. Also, the definition of an infectious disease did not include those with other diseases that might be considered infectious like chronic obstructive lung disease and asthma. However, those cases made up only around 5% of the cohort and, if included, did not change the models significantly (results not shown). Finally, the model intended for retrospective risk adjustment for setting comparisons should not be extrapolated to attempts to predict antibiotic use at admission. Nevertheless, the model that only included admission variables was also good (C-statistic > 0.8).

We conclude that adjustments for non-modifiable factors, such as the ones we have established, may increase confidence in observed surveillance results. Furthermore, nearly a quarter of the total antibiotic days were in patients with short hospital stays, antibiotic treatment over 7 days, and in those without a primary diagnosis of infection. Selecting those outcomes might aid in comparing internal medicine departments and focus audits and feedback interventions. The results need to be confirmed in other settings.

## Data Availability

The data are not publicly available but available on reasonable request after approval from the hospital.

## Table 1. Long description

A table comparing the proportion of various antibiotics administered to all patients and those treated with antibiotics. The table has 16 rows and 4 columns. The columns are labeled ‘Drug’, ‘N’, ‘All patients N=11,402 (%)’, and ‘Treated with antibiotics N=4,903 (%)’. The rows list different drugs and their corresponding values. Row 1: Other, 415, 3.6, 8.5. Row 2: Metronidazole, 1,036, 9.1, 21.1. Row 3: Carbapenem, 355, 3.1, 7.2. Row 4: Quinolone, 256, 2.2, 5.2. Row 5: Aminoglycosides, 229, 2.0, 4.7. Row 6: Penicillin, 157, 1.4, 3.2. Row 7: Beta lactams, 206, 1.8, 4.2. Row 8: Macrolide, 413, 3.6, 8.4. Row 9: Cephalosporins - other, 1,280, 11.2, 26.1. Row 10: Ceftriaxone, 2,840, 24.9, 57.8. Row 11: Chloramphenicol, 826, 7.2, 16.8. Row 12: Clindamycin, 540, 4.7, 11.0. Row 13: Colistin, 9, 0.08, 0.1. Row 14: Vancomycin, 120, 1.1, 2.4. Row 15: Piperacillin/tazobactam, 470, 4.1, 9.6. Row 16: Tetracycline, 629, 5.5, 11.5. Row 17: Reserve antibiotics, 1,015, 8.9, 20.7.

Navigate back to Table 1..

## Table 2. Long description

A table with 12 rows and 5 columns showing variables associated with antibiotic therapy. The columns are labeled Variable, N, N (%), OR, and 95% CI. The table includes variables such as Age years, Female, Previous hospitalization, Fever, Bedridden, Dementia, Permanent urinary Catheter, Hospital days, Infectious disease, and Department. Each row provides the count (N), percentage (N %), odds ratio (OR), and 95% confidence interval (95% CI) for each variable. Notable variables include Fever with an OR of 9.14, Infectious disease with an OR of 29.05, and Hospital days >7 with an OR of 4.67.

Navigate back to Table 2..

## Table 3. Long description

A table with 8 rows and 5 columns showing univariate analysis of laboratory tests associated with antibiotic therapy. The columns are labeled Variable, N, N (%), Odds ratio, and 95% CI. The rows list different variables such as Albumin, Creatinine, Glucose, WBC, Neutrophils, Hemoglobin, Platelets, and Positive urine culture. Each row provides the count (N), percentage (N %), odds ratio, and 95% confidence interval (CI) for each variable. For example, for Albumin (g/dL >= 3.5), N is 9,151, N (%) is 37.9, Odds ratio is 2.04, and 95% CI is 1.90-2.18. The table provides detailed statistical data on various laboratory tests and their association with antibiotic therapy.

Navigate back to Table 3..

## Table 4. Long description

The table presents data on factors associated with antibiotic treatment for the years 2020 and 2021, along with combined data for both years. It includes predictors such as infections, fever, length of stay (LOS) less than 3 days, LOS greater than 7 days, white blood cell count (WBC), neutrophils, platelet count, creatinine levels, and bacteriuria. The table also categorizes deciles of risk and provides H-L statistic values for each year and combined. The table has 15 rows and 4 columns. Column headers are Predictors, 2020, 2021, and Both years. Row labels include various predictors and deciles of risk. Each cell contains specific values related to the predictors and risk deciles for the respective years.

Navigate back to Table 4..

## Table 5. Long description

A table with predictors and their associated values for antibiotic therapy in 2020-2021. The table has 20 rows and 2 columns. The first column lists predictors such as hospitalization in the last 4 weeks, admission infectious diagnosis categories, bedfast status, dementia, fever, WBC, neutrophils, platelet, hemoglobin, albumin, and department. The second column shows the associated values for these predictors. Notable predictors include skin/soft issue infection with a value of 13.65, fever with a value of 5.15, and being bedfast with a value of 1.60. The table also includes deciles of risk with corresponding values and statistics like the Hosmer-Lemeshow statistic and C-statistic.

Navigate back to Table 5..

## Table 6. Long description

A table with 17 rows and 4 columns. The columns are labeled Predictors, Odds ratio, and 95 CI. The table lists various predictors of antibiotic therapy during hospitalization of 48 hours or less, along with their corresponding odds ratios and 95 percent confidence intervals. The predictors include age group, bedfast status, urinary catheter use, fever, admission infectious diagnosis categories (urinary tract, respiratory tract, skin/soft tissue, other), WBC, neutrophil percentage, glucose, creatinine, and albumin levels. The table also includes deciles of risk with the number of cases and total cases, along with their percentages. The Hosmer-Lemeshow statistic and C-statistic are provided at the bottom.

Navigate back to Table 6..

## Table 7. Long description

A table with 17 rows and 3 columns. The columns are labeled Predictors, Odds ratio, and 95% CI. The table lists various predictors such as Female, Previous hospitalization, Permanent catheter, Bedfast, Fever, UTI admission diagnosis, WBC, Neutrophils proportion, Hemoglobin, Platelet counts, and Department B. Each predictor has an associated odds ratio and a 95% confidence interval. The table also includes deciles of risk with corresponding values and statistics like the Hosmer-Lemeshow statistic and C-statistic.

Navigate back to Table 7..
